# Argonaute-2 slicing of a domesticated retrotransposon maintains proteostasis and prevents lethal skeletal muscle defects

**DOI:** 10.64898/2026.05.22.727060

**Published:** 2026-08-18

**Authors:** Nickolas Almodovar, Caroline Dunn, Benjamin Kleaveland

## Abstract

Argonaute (AGO) proteins associate with small guide RNAs to bind and repress mRNA targets. AGO2 is the primary AGO slicer in mammals, cleaving RNAs that base-pair extensively to its guide RNA. Disrupting the slicing activity of AGO2 in mice causes neonatal lethality, however, the specific cell types and substrates that contribute to this lethality are not known. Through a combination of genetic, histologic, and molecular approaches, we identify retrotransposon-like-1 ( *Rtl1* ) as a key AGO2 slicing substrate in placental endothelium and skeletal muscle, and show that AGO2 slicing in skeletal muscle, but not endothelium, is required for postnatal viability. Loss of AGO2 slicing causes cell-autonomous, pathologic changes in skeletal muscle, characterized by larger fibers, increased central nuclei, prominent central clearings, and induction of transcripts associated with heat shock proteins. Induced expression of RTL1, a domesticated retrotransposon-derived protein that can self-assemble into viral-like capsids, activates a similar heat shock protein response in cultured myoblasts, underscoring the importance of limiting excess RTL1 during muscle development. Taken together, our findings demonstrate a critical role for AGO2 slicing in skeletal muscle development that is, at least in part, due to its repression of a domesticated retrotransposon.

## Full Text Availability

The license terms selected by the author(s) for this preprint version do not permit archiving in PMC. The full text is available from the preprint server.

